# University College London/University of Gothenburg PhD course “Biomarkers in neurodegenerative diseases” 2019—course organisation

**DOI:** 10.1186/s13195-020-0583-z

**Published:** 2020-02-04

**Authors:** Ayesha Khatun, Ross W. Paterson, Michael Schöll

**Affiliations:** 1 Dementia Research Centre, Queen Square Institute of Neurology, University College London, London, UK; 2 Wallenberg Centre for Molecular and Translational Medicine and the Department of Psychiatry and Neurochemistry, University of Gothenburg, Gothenburg, Sweden; 3 Department of Clinical Physiology, Sahlgrenska University Hospital, Gothenburg, Sweden

**Keywords:** Neurodegenerative diseases, Biomarkers, Tau, Amyloid, Positron emission tomography, Magnetic resonance imaging, Cerebrospinal fluid, Blood biomarkers

## Abstract

Biomarkers are increasingly employed for effective research into neurodegenerative diseases. They have become essential for reaching an accurate clinical diagnosis, monitoring disease, and refining entry criteria for participation in clinical treatment trials, and will be key in measuring target engagement and treatment outcome in disease-modifying therapies. Emerging techniques and research combining different biomarker modalities continue to strengthen our understanding of the underlying pathology and the sequence of pathogenic events. Given recent advances, we are now at a pivotal stage in biomarker research. PhD students working in the field of neurodegenerative disease require a working knowledge of a range of biomarkers available and their limitations, to correctly interpret scientific literature and to design and conduct successful research studies themselves. Here, we outline the University College London/University of Gothenburg “Biomarkers in neurodegenerative diseases course”, the first initiative of its kind aimed to bring together both experts and PhD students from all areas within the field of neurodegeneration, to provide comprehensive knowledge of biomarker research for the next generation of scientists.

## Introduction

This paper is an introduction to a series of reviews based on the course as below:

- Paper 1: University College London/University of Gothenburg course “Biomarkers in neurodegenerative diseases 2019”—course organisation (Ayesha Khatun et al.)
- Paper 2: The utility of biomarkers for neurodegenerative diseases: clinical and research perspectives (Alexander J Ehrenberg et al.)
- Paper 3: Fluid biomarkers in neurodegenerative diseases: perspectives from the University College London/University of Gothenburg course (Pawel Obrocki et al.)
- Paper 4: Imaging biomarkers in neurodegeneration: Current and future perspectives (Peter NE Young et al.)

The field of biomarker research in neurodegenerative diseases continues to rapidly grow to include an increasing number of modalities and techniques. These provide complementary strengths in identifying neurodegenerative diseases early, reaching consensus diagnosis in both research and clinical settings, and will be key in refining clinical trial inclusion criteria, disease monitoring, measuring target engagement in disease-modifying therapies as well as in assessing associations between biomarker-defined pathology with clinical endpoints.

The first “Biomarker in neurodegenerative diseases” course was held at the University of Gothenburg (UGOT) in 2018 after local researchers Michael Schöll and Henrik Zetterberg who work in highly complementary fields within neurodegeneration identified a lack of international doctoral-level education in multimodal biomarker research. They considered this an opportunity to bring together their wider network of colleagues into a course expressing the breadth of biomarker practice in neurodegeneration, with an emphasis on Alzheimer’s disease (AD).

The outline of the course is published to serve as an example for other course organisations in the field as well as an introduction to a series of biomarker reviews written by the course delegates.

The second edition of the course was organised as a collaborative initiative between University College London (UCL) and UGOT in April 2019, and we intend to continue offering the course alternating between both sites.

The overall course aim is to provide PhD students from different fields with basic and practical knowledge about both bodily fluid- and brain imaging-derived biomarkers for neurodegenerative diseases. Students are expected to achieve a broad understanding of disciplines, including advanced brain imaging, and neurochemistry technologies used as research and clinical tools including:

- Neurochemistry
- Biomarkers in cerebrospinal fluid (CSF)
- Biomarkers in blood
- Structural and functional magnetic resonance imaging (MRI)
- Positron emission tomography (PET)

UGOT and UCL are leading international centres with complementary expertise in fluid and imaging biomarkers. We anticipated that this collaboration would strengthen the quality of biomarker training across both institutions and beyond, as well as foster new research collaborations and provide delegates with outstanding networking opportunities internationally.

Following completion of the course, delegates were expected to be able to:

- Explain basic concepts in fluid- and image-based biomarker research
- Describe how different biomarkers relate to each other in a temporal, pathogenic, and regional (anatomical) context of different neurodegenerative diseases
- Conduct the planning of a project within their own area of research where the use of the discussed biomarkers is explained
- Use basic tools to evaluate biomarker data
- Interpret biomarker profiles in different neurodegenerative diseases
- Understand when biomarkers/methods can and cannot be applied
- Analyse the predictive value of the respective biomarkers in different conditions

## Methods: overview of the biomarker course

**Fig. 1:**
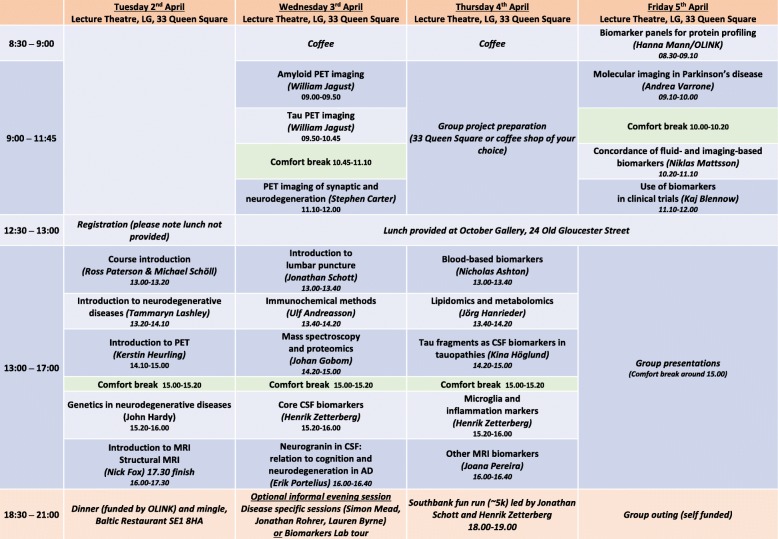
UGOT/UCL Biomarkers in Neurodegeneration 2019 course timetable

This year’s course at UCL ran over 4 days (Fig. 1) in April 2019. A total of 52 students including international delegates took part, with a further 23 on the waiting list.

Delegates included a mixture of students from clinical medical research background (50%), natural sciences/medicine (41%), and health care sciences (2.9%). The course was aimed at international PhD students working broadly in the field of biomarkers in neurodegeneration. Students who were unable to register for the 2019 course were given priority to register for the 2020 course in April. In addition to the lectures, a self-directed group exercise was assigned with groups of four to six delegates. Based on knowledge acquired during the course, delegates were asked to develop a research project proposal and subsequently delivered a 15-min study proposal presentation in front of an expert panel. We collected prospective information from delegates on their area of research and split groups according to expertise to try and achieve a degree of balance. We also aimed to mix delegates from the same institutions and to balance gender.

We also focussed on mechanisms for enhancing the student experience through a buddy scheme and the organisation of evening social events (Fig. 1). The buddy scheme identified delegates within the UK and allocated them up to three colleagues from outside the UK who could contact them for advice on travel/accommodation and life in London.

At the end, students were asked to fill in a feedback questionnaire to help us improve future courses (Table 1). Overall, students were satisfied with the course (97%) and felt that the aim of the course was clear and fulfilled, with administration of the course well carried through (94%).

**Table 1:** Course feedback

Questions	Overall agreement (%)
I am generally satisfied with the course and what I learned	97
The aim of the course was clear	94
The aim of the course was fulfilled	94
The administration of the course was well carried through	97
The teachers in general had good pedagogic abilities and showed interest in student learning	99
The lectures were excellent	97
The course literature was relevant in relation to aims and purposes of course	88
The examination was relevant in relation to the aims and purposes of the course	68
The balance between theoretical lecture, group practices and discussions was good	95
The content of the course is relevant to my research	83
I would recommend this course to doctoral students in my field of research	91
The allocation of higher education credits was reasonable in relation to the workload	82

Students felt that the lecturers in general had good teaching skills and showed interest in student learning (99%) and that lectures themselves were excellent (97%).

Students were provided with course literature and pre-reading materials which they felt were relevant in the relation to the aims (88%), and 68% of students felt that the examination was relevant in relation to the aims and purpose of the course; however, no alternative examinations were suggested.

Ninety-five percent of students agreed that the balance between lectures and the practical elements was good. Eighty-three percent felt that the course content was relevant to their individual research, and 91% agreed that they would recommend the course to other doctoral students.

We will aim to revise the examination component of the course as it had received the lowest score at 68%.

The following topics were covered (in order of delivery):

- *Introduction to neurodegenerative diseases pathology—Dr Tammaryn Lashley*

A definitive diagnosis for a neurodegenerative disease can only be given, at present, by post-mortem examination of the brain. This lecture focussed on the work at the Queen Square Brain Bank where underlying pathological features are observed macroscopically and microscopically. Alzheimer’s disease (AD) is diagnosed using standard criteria based on the presence and spread of extracellular amyloid-β (Aβ) and intracellular tau. Frontotemporal dementia is a heterogeneous group of diseases that clinically overlap, and the underlying pathological hallmarks could be one of three major proteins: tau in FTLD-Tau, TDP-43 in FTLD-TDP, and FUS in FTLD-FUS. Here, we go through the major pathological hallmarks used to identify and diagnose the different diseases. We also highlight that these neurodegenerative diseases can co-exist [1–6].

- *Introduction to PET imaging—Dr Kerstin Heurling*

This lecture discussed PET as a molecular imaging method, based on the detection of disintegration of short-lived radioactive isotopes incorporated in pharmacological molecules (known as PET tracers or ligands) with affinity to a physiological target, such as beta-amyloid [7]. Medical images are created, showing the distribution of the radioactivity, corresponding to the amount of target in the tissue imaged. Absolute quantification of the tracer binding requires long scanning times and plasma sampling, but semi-quantitative measures such as the ratio of radioactivity concentration in a region of interest (ROI) relative to one without target (a measure known as standardised uptake value ratio (SUVR)) is often sufficient [8].

- *Genetics—Professor John Hardy*

This talk discussed the genetic analysis of Alzheimer’s disease, Parkinson’s disease, and tauopathies. It pointed out that in all genetic cases overexpression of the primary protein deposited gives rise to disease: APP duplication causes AD, SNCA duplication causes PD, and MAPT duplications cause tauopathies. Other Mendelian causes also often lead to overproduction of the deposited protein. This talk discussed how many of the risk genes for “sporadic” late onset disease are involved in the clearance of these same proteins. In the case of amyloid, this clearance is largely microglial; in the case of synuclein, the clearance is largely lysosomal; and in the case of tau, the clearance is at least partly through the ubiquitin proteasome. Thus, there is a consistency in the pathogenesis of these diseases. Overproduction of these proteins and a problem in clearance are the general causes of these diseases [9].

- *Introduction to MRI—Professor Nick Fox*

This talk focussed on structural MRI (sMRI) in both clinical and research settings. It provided an overview of the principles of image formation and how what can be “seen” is determined by image resolution and contrast. The basic physics of MRI was introduced, and elements of a modern MR scanner were briefly reviewed. It also discussed how sMRI remains the mainstay of clinical imaging in dementia. The lecture also looked at the evolving roles of clinical imaging: which had moved from a purely exclusionary approach to one where one gains positive support for a particular diagnosis [10–12].

- *Amyloid and tau PET imaging—Professor William Jagust*

Measurement of aggregated proteins in the brain in ageing and dementia has become a standard approach to characterising research participants in clinical, translational, and therapeutic studies. This lecture reviewed the approaches to biomarker characterisation using PET scanning with ligands that bind to the key pathological hallmarks, aggregated amyloid-β (Aβ) and tau proteins. This approach has helped to establish a new framework for research classification [13] and contributed to our understanding of the pathophysiology of AD [14]. The lecture further discussed the dynamics between these measures, age and cognition.

- *PET imaging of synaptic and neurodegeneration—Dr Stephen Carter*

The AD biomarker model [15] indicates neurodegeneration occurs late in the disease process. The PET biomarkers [18F] FDG and [11C]UCB-J measure neurodegeneration and changes in synaptic integrity in vivo. The well-established and most used PET ligand [18F] FDG measures reductions in brain glucose metabolism whereas novel ligand [11C]UCB-J assesses reduced synaptic density. This lecture summarised studies for each biomarker, including how imaging data is typically processed, analysed, and interpreted [15–17].

- *Introduction to lumbar puncture—Professor Jonathan Schott*

Ultimately, any CSF biomarkers for dementia need to be applied in clinical practice, and the results interpreted on individual patient basis. In this lecture, the case for the use of molecular diagnostics in the differential diagnosis of dementia—and in particular a positive diagnosis of AD—were made, alongside a review of the core CSF biomarkers currently in use, proposed “good use” criteria, the practicalities of CSF sampling and storage, and interpretation of results including the advantages and limitations of using cut-points. Finally, the diagnostic role of CSF biomarkers for individual patients was illustrated using a number of patient case studies [18–20].

- *Immunochemical methods—Dr Ulf Andreasson*

Immunochemical methods are widely used both for established biomarkers and in the search for new ones. The lecture covered the principles of different methods and platforms, including ultra-sensitive ones [21], as well as multiplex methods. Some possible sources of interference were discussed, and the importance of assessing the performance by technical validation of an assay was stressed [22].

- *Mass spectrometry and proteomics—Dr Johan Gobom*

This lecture focussed on mass spectrometry, an analytical technique used to measure the molecular mass of a broad range of analytes, ranging from small volatile molecules to large biomolecules. The ability to identify and quantify large numbers of proteins and peptides in biological samples by mass spectrometry has given rise to the research field proteomics, which is applied in many research areas, such as neuroscience [23]. Performing proteomic analysis of clinical samples—clinical proteomics—can be used to identify new biomarkers [24]. Clinical proteomics is still a young field; while clinical proteomic studies have resulted in the identification of hundreds of new candidate CSF markers of Alzheimer’s disease [25], the majority remains to be validated [24].

- *Core CSF biomarkers—Professor Henrik Zetterberg*

This lecture revisited the evidence on what the standard CSF biomarkers for AD may represent. It concluded that the CSF concentration of the 42 amino acid-long isoform of Aβ (Aβ42) correlates inversely with plaque pathology in the brain and that the ratio of Aβ42 to Aβ40 (CSF Aβ42/Aβ40 ratio) corrects for inter-individual differences in amyloidogenic processing of the amyloid precursor protein (APP), resulting in an even more accurate plaque pathology test with 90–95% concordance with amyloid PET. CSF total and phosphorylated tau (T-tau and P-tau, respectively) are not direct but rather predictive markers of AD-type neurodegeneration and tangle pathology. Recent stable isotope kinetics studies in humans and human-derived cell models [26], as well as earlier studies in mouse models of AD [27], suggest that neurons affected by Aβ pathology phosphorylate and secrete more tau into the CSF in an active process. Such neurons may eventually degenerate and develop tangle pathology, explaining why CSF tau and tau PET correlations appear in late-stage disease but are difficult to discern in pre-dementia disease stages.

- *Fluid-derived biomarkers for inflammation—Professor Henrik Zetterberg*

The lecture discussed the small but significant changes in CSF that are suggestive of microglial and astrocytic activation (CSF sTREM2 and YKL-40, respectively) in AD. CSF interleukin and cytokine concentrations are most often relatively normal. Classical neuroinflammation changes in CSF (e.g. increased CSF cell counts and CSF/serum albumin ratio) should raise suspicion on a primary neuroinfectious or inflammatory disease. For example, neuroborreliosis should be excluded [28].

- *Use of biomarkers in clinical trials—Professor Henrik Zetterberg/Professor Kaj Blennow*

The lecture looked at several potential uses of biomarkers in clinical trials, mainly for AD. Biomarkers may be used to diagnose and exclude patients with neuroinflammatory and neuroinfectious conditions. Specific imaging or fluid biomarkers for AD pathology may be used as supporting diagnostic markers in the clinic and as additional inclusion criteria in studies of anti-AD drugs. Depending on the mechanism of action of the drug, specific imaging or fluid markers may be used for drug effect monitoring. There are also a number of downstream markers that a disease-modifying drug is expected to have an effect on, irrespective of the mechanism of action. If a drug is effective at slowing neurodegeneration, CSF neurofilament light and/or T-tau concentrations should decrease and MRI changes should progress slower. Finally, it was discussed that biomarkers could be used as safety markers in clinical trials (e.g. MRI for ARIA and CSF cell count and CSF/serum albumin ratio for treatment-induced neuroinflammation) [29].

- *Neurogranin in CSF relation to cognition and neurodegeneration in AD—Dr Eric Portelius*

The lecture covered what we have learned so far about the post-synaptic protein neurogranin. Neurogranin is highly expressed in the brain, especially in the cortex, hippocampus, and amygdala, and several studies have shown that the cerebrospinal fluid levels of the protein are increased in AD patients compared to healthy controls. In addition, increased levels of neurogranin seem to be specific for AD since the levels seem not to be increased in other diseases affecting the central nervous system [30].

- *Blood-based biomarkers—Dr Nicholas Ashton*

The rapid advancement of ultra-sensitive platforms for protein analysis has enabled the investigation of neuropathological proteins to be measured readily in blood samples. This has tremendous implications for the clinical management and patient monitoring in therapeutic trials of neurodegenerative diseases. This lecture began with an overview of the previous two decades of efforts in the search for a blood-based biomarker for AD, focusing on the challenges and limitations that have been encountered. Building on lectures covering the advancements in immunological assays and mass spectrometry, it discussed the latest research in how amyloid and neurofilament light proteins measured in blood predict cognitive decline and related imaging measures [30–33].

- *Lipidomics and metabolomics—Dr Jörg Hanrieder*

Imaging mass spectrometry is an emerging chemical imaging modality allowing comprehensive delineation of spatial distribution pattern of biochemical species in situ, including metabolites, neurotransmitters, lipids, neuropeptides, and small proteins [34]. The lecture covered basic principles of imaging MS modalities along with the more established “omics” method paradigms based on tissue and body fluid extraction and liquid chromatography and mass spectrometry. A particular focus lies here on using these novel tools for probing neurodegenerative disease pathology both in human brain as well as in animal model systems [35].

- Tau species as biomarkers in tauopathies—Dr Kina Höglund

The role of CSF total tau was discussed across both AD and other tauopathies. The value of tau assays to measure tau and p-tau was examined, looking at their diagnostic significance in primary and secondary tauopathies. The lecture also highlighted the complexity and heterogeneity of tau in CSF, where several tau fragments coming from proteolytic cleavage are present, which are not measured by traditional assays [36, 37]. Results from novel assay directed to N-terminal and C-terminal protein fragments were also presented, suggesting that their implementation in research settings could improve both the diagnostic profiling of tauopathies and the understanding of the disease aetiology.

- *Other MRI biomarkers—Dr Joana Pereira*

This lecture introduced the students to the growing field of brain connectomics and the methods that can be used to assess functional and structural brain connectivity on functional magnetic resonance imaging and diffusion tensor imaging. It also provided a detailed description of the properties of the human brain connectome using concepts from graph theory [38]. For instance, the small-worldness is a property that can be used to characterise the balance between long-distance and short-distance brain connectivity whereas the modularity defines how well the whole brain network can be subdivided into subnetworks which generally overlap with well-known brain systems. These network properties and many others can be used to reveal fundamental aspects of normal brain organisation and highlight important aspects of underlying brain pathology in neurodegenerative disorders such as Alzheimer’s disease. For example, there is increasing evidence that brain regions with a higher number of connections show a greater disease-related vulnerability and may constitute important pathways for the spread of brain pathology such as amyloid-β and tau. Thus, network models could be used to monitor disease progression along connectional pathways and improve the early diagnosis of neurodegenerative diseases.

- *Biomarker panels for protein profiling—Ms Hanna Mann*

There are many different approaches to identify potential biomarkers, and genomic technologies have historically led the way, but this does not provide us with the whole story. This presentation focussed on protein biomarker discovery using the Proximity Extension Assay (PEA) technology from Olink Proteomics, making it possible to analyse over 1100 protein biomarkers using very small volumes of plasma, serum, or CSF and how multi-omics approaches can advance precision medicine [39].

There was a specific focus on the two Neurology protein panels and examples from scientific publications in the neurology field. Multi-omics strategies and large data sets present new opportunities but also challenges when designing and implementing biomarker studies, and this lecture included an introduction to different study design scenarios.

- *Molecular imaging in Parkinson’s disease—Dr Andrea Varrone*

This lecture provided an overview of the neuropathological features of movement disorders and Parkinson’s disease and its relevance to imaging. Three major topics were discussed: protein misfolding, neurodegeneration, and microglia activation. Emphasis was given to the description of how PET can be applied to image those pathological features in vivo.

The first part of the lecture discussed the status of PET imaging of alpha-synuclein, tau and amyloid-β. At present, the development of a PET tracer for imaging alpha-synuclein is still a major challenge, mainly due to the fact that high affinity and selectivity are required to be able to image Lewy pathology in vivo. More data are available on tau and amyloid imaging in Parkinson’s disease and related movement disorders. A systematic review has shown that the prevalence of amyloid-positive cases is approximately 70% in case of dementia with Lewy bodies (DLB) and approximately 1/3 in cases of PD with dementia [40]. Tau accumulation increases in the spectrum of Lewy body disease and the load of tau increases together with burden of amyloid [41]. In DLB, amyloid burden tends to be lower than in AD. In the DLB cases that are amyloid positive, there is a greater involvement of primary cortices and less prominent involvement of the temporal cortex [41].

Specific areas of tau accumulation have been reported in patients with progressive supranuclear palsy, with more prominent involvement of the globus pallidus, substantia nigra, and dentate nucleus of the cerebellum [42]. Extended involvement of the white matter has been associated with increase of severity of motor symptoms [43]. In corticobasal syndrome, tau accumulation has been observed in the white matter with prevalent involvement of the precentral gyrus [44]. First-generation tau tracers used so far do present some limitations, related to the presence of off-target binding in some areas, such as the basal ganglia, that are primarily affected by the pathology. The introduction of second-generation tau radioligands might be useful to evaluate more specifically the patterns of tau observed in PSP and CBS.

The second part of the lecture has focussed on the review of the tracers available to study nigrostriatal dopaminergic degeneration in PD. The current view is that alpha-synuclein accumulation is associated with synaptopathy leading to early degeneration in the synaptic terminals, followed by later degeneration in the axons and cell bodies of the substantia nigra. The development of ^18^F-FE-PE2I as DAT tracer has provided for the first time the possibility to study in vivo the entire nigrostriatal system [45]. In early PD patients, the DAT along the axons and in the substantia nigra is relatively preserved compared with the striatum, suggesting that most of the cell bodies and projections are still preserved in the early stages of the disease and their function might be restored with proper treatment.

The third part of the lecture has reviewed the status of imaging of microglia activation in PD and related disorders. Initial studies with the first-generation TSPO radioligand [^11^C]PK11195 have reported increased binding to TSPO in PD, as well as in MSA, PSP, and CBD. Subsequent studies in PD patients with second-generation TSPO radioligands have not replicated the initial findings [46, 47]. Therefore, it is still controversial whether it is possible to image microglia activation in PD, considering the complexity of the process and the different expression in relation to the stage of the disease.

- *Concordance of fluid- and imaging-based biomarkers—Dr Niklas Mattsson*

CSF biomarkers and PET have similar and high accuracy to detect Aβ pathology in vivo, especially at the dementia stage of AD [48]. However, some studies suggest that reductions in CSF Aβ_1–42_ may precede increased uptake of Aβ PET [49, 50]. This discrepancy has been used to study the earliest regions affected by Aβ accumulation in AD [51] and to construct an in vivo amyloid PET staging system to monitor the spatiotemporal spread of Aβ [52]. For tau, most CSF and PET measures are only moderately correlated which each other [53, 54]. One possible explanation for this is that CSF tau measures change early and reflect the presence of a disease state of AD, while tau PET changes progressively over the course of the disease and is more related to the disease stage. Available data suggest that CSF tau may represent changes in the soluble metabolism of tau in response to beta-amyloid pathology, which precede the deposition of tau aggregates that is visualised by tau PET [55, 56].

### Course organisation

Dr Michael Schöll (University of Gothenburg and University College London) is an Associate Professor in Molecular Medicine (UGOT) and a Principal Research Fellow (UCL) with a focus on neuroimaging. He started the course in Gothenburg in 2018 in collaboration with Professor Henrik Zetterberg. His research aims to use neuroimaging and neuropathological changes to assess neurodegeneration in comparison to healthy ageing.

Dr Ross W Paterson (University College London) is a Senior Research Fellow and Honorary Consultant Neurologist at the Dementia Research Centre at UCL with interests in CSF biomarkers in neurodegenerative diseases and young onset and rapidly progressive dementias.

Ayesha Khatun (University College London) is a Study Coordinator for the local Familial AD study at The Dementia Research Centre, with an MSc in Neuroscience and an interest in fluid biomarkers in dementia.

### Future directions

The course will be offered again at the University of Gothenburg in April 2020. Interested PhD students are invited to contact a.khatun@ucl.ac.uk or eva.bringman@gu.se and visit the following website for more information: https://wcmtm.gu.se/research-groups/scholl/courses.

Future revisions of the courses aim to include biomarkers encompassing an even wider reach of neurodegenerative diseases. We further aim to include a biomarker statistics workshop and a scientific paper writing class, as well as focus on emerging novel tools and techniques that probe alternative neurophysiological data and novel biomarkers.

## Conclusions

Given the developments in disease-modifying drugs for neurodegenerative diseases, especially AD, there is a clear need for educational efforts delivering the latest research from both the fields of imaging- and fluid-derived biomarkers for neurodegenerative diseases. The UGOT/UCL course organised in 2018 and 2019, with plans to offer the course on an annual basis, aims to provide a comprehensive learning and networking platform for international doctoral-level (and above) biomarker researchers. We believe that bringing together prominent lecturers and the next generation of researchers in the biomarker field will result in informed and orchestrated scientific endeavours as well as in creating an open international scientific community.

## Data Availability

This review does not contain any analysable data. All sources cited in this paper are publicly available.

## Abbreviations

Aβ: Amyloid-β
AD: Alzheimer’s disease
APP: Amyloid precursor protein
ARIA: Amyloid-related imaging abnormalities
CSF: Cerebrospinal fluid
FTLD: Frontotemporal lobar degeneration
FUS: Fused in sarcoma
MAPT: Microtubule-associated protein tau
MRI: Magnetic resonance imaging
PD: Parkinson’s disease
PET: Positron emission tomography
ROI: Region of interest
SUVR: Standardised uptake value ratio
TDP-43: TAR DNA-binding protein 43
